# The relationship between inflammatory response markers and the prognosis of non-muscle invasive bladder cancer and the development of a nomogram model

**DOI:** 10.3389/fonc.2023.1189086

**Published:** 2023-06-29

**Authors:** Xinping Yi, Jiangchuan Pi, Chuan Liu, Yongjiang Xiong, Jiaji Liu, Wenyu Fu, Lanxi Wang, Tao Zhao

**Affiliations:** ^1^ Department of Urology, Yongchuan Hospital of Chongqing Medical University, Chongqing, China; ^2^ Department of Urology, Chengdu Second People’s Hospital, Chengdu, China; ^3^ Department of Urology, The Second Affiliated Hospital of Chongqing Medical University, Chongqing, China; ^4^ Ophthalmology, The Second Affiliated Hospital of Chongqing Medical University, Chongqing, China

**Keywords:** inflammatory factors, prognostic risk factors, tumor recurrence, Kaplan-Meier survival, intravesical instillation

## Abstract

**Purpose:**

Patients with non-muscle invasive bladder cancer (NMIBC) have a high possibility of recurrence after surgery. We aimed to assess the factors associated with tumor recurrence and to construct a nomogram model that can contribute to personalized treatment plans of each patient.

**Methods:**

496 patients with primary bladder cancer (BC) from 2 centers were retrospectively analyzed. Preoperative neutrophil/lymphocyte ratio (NLR), platelet/lymphocyte ratio (PLR), systemic immune-inflammation index (SII), and traditional clinical parameters were collected, then using univariate and multivariate Cox regression analysis to find out the independent risk factors associated with tumor recurrence among them, and then these independent factors were incorporated into the nomogram model. The internal calibration curves and the external calibration curves were used to verify their usefulness.

**Results:**

In the training cohort, 150 patients (43.1%) experienced recurrence. After Cox regression analysis, the independent risk factors affecting recurrence-free survival (RFS) were tumor grade, immediate postoperative instillation therapy (IPPIT), NLR, and SII. These factors were used to construct a model to predict RFS 1, 2, 3, and 5 years of NMIBC patients after surgery. And then, we found that the constructed model outperforms the conventional model in terms of accuracy and predictability, the results were verified by statistical tests.

**Conclusion:**

Preoperative inflammatory response markers have a predictive value for postoperative recurrence in patients with NMIBC. The constructed nomogram model can be helpful in guiding personalized clinical evaluation and subsequent treatment.

## 1 Introduction

Bladder cancer (BC) is the second most commonly diagnosed urological malignancy after prostate cancer. Worldwide, its incidence ranks 10th among malignant tumors, and the incidence has steadily increased recently, causing a serious impact on patient survival (1, 2). According to clinical progression and prognosis, bladder malignancies can be classified as either muscle-invasive bladder cancer (MIBC) or non-muscle-invasive bladder cancer (NMIBC), with NMIBC accounting for approximately 70% of cases. More than 90% of all pathological types are urothelial carcinoma (UC), so we will only discuss this.

Currently, the main treatment modality for NMIBC is transurethral resection of bladder tumor (TURBT) with or without intravesical instillation, depending on the patient’s risk stratification. Despite complete resection and adjuvant intravesical instillation, disease recurrence occurs in up to 70% of these patients (3). Therefore, in the era of personalized treatment, accurate prediction of recurrence and appropriate follow-up therapy have become indispensable steps for clinicians in the postoperative management of bladder cancer patients. Previously, models for predicting NMIBC prognosis have relied on pathological features such as T-stage, grading, tumor diameter, recurrence rate, and concomitant carcinoma *in situ* (CIS). Although these traditional clinical parameters have been used to predict recurrence and progression in patients with BC, their accuracy still needs to be improved (4, 5). Recently, several prognostic models and biomarkers have been investigated as predictors of BC recurrence to guide clinical decision-making and patient counseling (6, 7). In fact, most biomarkers are not routinely used in clinical practice, partly due to high costs and lack of standardization. Therefore, the use of an inexpensive and clinically accessible indicator as a prognostic marker and thus the creation of a model to predict recurrence has recently become a hot research topic.

Recently, the interaction between the inflammatory response of the body and the development of cancer has received increasing attention, as the inflammatory response can stimulate cellular oxidative damage and gene mutation and provide a favorable microenvironment for the growth of tumor cells, which in turn accelerates tumor cell proliferation, invasion, and metastasis (8, 9). Similarly, inflammatory responses in the tumor microenvironment (TME) play a key role in bladder tumorigenesis, proliferation, progression, and metastasis (10, 11). The ability of inflammatory response markers to predict cancer prognosis has been extensively researched for the neutrophil-to-lymphocyte ratio (NLR), platelet-to-lymphocyte ratio (PLR), monocyte-to-lymphocyte ratio (MLR), and systemic immune-inflammation index (SII) (12–14). However, few studies have used them to predict the prognosis of patients with NMIBC after surgery and to construct nomogram models. Therefore, constructing an accurate prognostic model can help clinicians personalize treatment plans for patients to reduce the recurrence rate of bladder cancer and improve their quality of life.

## 2 Materials and methods

### 2.1 Study population

Our study included Primary BC patients from *yongchuan hospital of Chongqing Medical University*, and *the second affiliated hospital of Chongqing Medical University*, who were treated by TURBT from 2012 to 2022. The inclusion criteria were as follows: 1. histopathological diagnosis of NMIBC; 2. no history of preoperative or postoperative pelvic radiotherapy; and 3. patients with complete clinical, laboratory, and follow-up data. The exclusion criteria were as follows: 1. the surgery did not correspond to the standard TURBT procedure, or patients received two or more bladder tumor resections or total or subtotal bladder resections; 2. concomitant systemic autoimmune diseases, blood diseases, and other diseases affecting blood inflammatory markers; 3. bladder carcinoma *in situ* (CIS): excluded due to poor differentiation, high risk of muscle invasion and high-grade aggression; 4. Types with variant histologies (VHs) include squamous, glandular, micropapillary, nested, and so on; they affect the patient’s disease-free survival (DFS) and cancer-specific survival (CSS) (15); 5. those with other malignant tumors in combination; and 6. those without regular follow-up or with short follow-up (less than 3 months).

### 2.2 Data collection and pathological evaluation

Clinical, histopathological, and demographic characteristics of the patients and hematological indicators within 2 weeks before surgery were obtained from the electronic medical record system. NLR was defined as the ratio of neutrophil count to lymphocyte count, PLR was the ratio of platelet count to lymphocyte count, and SII was the ratio of the product of neutrophil count and platelet count to lymphocyte count. According to previously published studies, the threshold values for high NLR, PLR, and SII were set at 2.5, 150, and 525, respectively (13, 16). The TNM staging method published by the Union for International Cancer Control (UICC) was used to stage the disease; meanwhile, the WHO bladder cancer grading system (2004/2016) was used for pathological grading (17).

### 2.3 Management and follow-up

All patients received complete TURBT. The postoperative chemotherapy regimens were determined according to international guidelines, the patient’s condition, and what was observed intraoperatively. The chemotherapy regimen included immediate postoperative instillation therapy (IPPIT) (within 24 hours of surgery), early postoperative instillation chemotherapy, which was conducted once a week (4–8 weeks after surgery), and continuous bladder instillation chemotherapy, which was conducted once a month (lasting for 6–12 months) immediately after the completion of early instillation chemotherapy. According to international guidelines, all patients in the low-risk group completed only early postoperative instillation chemotherapy, and the middle–high risk patients completed early postoperative instillation chemotherapy and subsequent continuous instillation chemotherapy (18). According to the EAU recommendations, all patients were monitored. Typically, patients underwent physical examinations, cytology tests, and cystoscopies every three months for the first two years, every six months from year two to year five, and then once a year (3). The gold standard for confirming the diagnosis of recurrence is histopathology. Recurrence-free survival (RFS) was defined as the time from TURBT to the first evidence of either recurrence or progression, cancer-related death, or final follow-up.

### 2.4 Statistical analysis

The statistical analysis was performed using SPSS 27.0 and R 4.2.1 software. t tests and rank sum tests were employed to analyze continuous variables, while Fisher exact tests and chi-square tests were applied to categorical variables. To examine the risk factors affecting the prognosis, the Cox regression model (SPSS software) was applied. Differences between groups were assessed with the log-rank test. All of the above tests were considered statistically significant with differences at P < 0.05.

Patients from Yongchuan Hospital of Chongqing Medical University were designated as the training cohort, and the factors associated with recurrence were included in the univariate Cox regression for analysis. Then, the statistically significant indicators were included in the multivariate Cox regression analysis, and finally, the prognostic independent risk factors were obtained. These prognostic independent risk factors were constructed into the nomogram model using the R software to predict patients’ RFS at 1, 2, 3, and 5 years postoperatively. Calibration curves were then used for internal and external validation. The optimal threshold of 1-year RFS for patients was determined using the receiver operating characteristics (ROC) curve and Youden index ([sensitivity + specificity] − 1), and the patients were divided into high and low RFS groups based on this threshold. The survival curves of the two groups were plotted using the Kaplan–Meier curve, and the nomogram was validated using Harrell’s concordance index (C-index). To further validate the predictive efficiency of this model, we will compare the C-index of the different models by comparing this model with the traditional model.

## 3 Results

### 3.1 Clinicopathological characteristics of the patient

This study finally included 348 patients from *yongchuan hospital of Chongqing Medical University* as the training cohort and 148 patients from *the second affiliated hospital of Chongqing Medical University* as the external validation cohort. Overall, the median age of the patients was 69 years (range: 23–90 years), and the median follow-up time was 55 months (range: 3–125 months). Twenty-four patients (4.8%) died from recurrence or metastasis of bladder cancer. In the training cohort, 150 (43.1%) patients experienced recurrence, with a median follow-up of 55 (4–125) months and a median RFS of 25 (4–125) months; in the validation cohort, 58 (39.2%) patients experienced recurrence, with a median follow-up of 60 (4–118) months and a median RFS of 30 (4–116) months. The patients’ basic clinical information from the training group and validation group are listed in **Table 1**. The basic data did not significantly differ between the two groups (P>0.05).

**Table 1:** Basic clinical data of the training cohort and the validation cohort.

Variables	Level	Number of patients (%)	
		Training cohort 348	Validation cohort 148
**Gender**	Male	274 (78.7)	110 (74.3)
	Female	74 (21.3)	38 (25.7)
**Age (years)**	<65	137 (39.4)	53 (35.8)
	≥65	211 (60.6)	95 (64.2)
**Hypertension**	No	229 (65.8)	101 (68.2)
	Yes	119 (34.2)	47 (31.8)
**Diabetes**	No	291 (83.6)	125 (84.5)
	Yes	57 (16.4)	23 (15.5)
**Smoking history**	No	146 (42.0)	74 (50.0)
	Yes	202 (58.0)	74 (50.0)
**T category**	Ta	220 (63.2)	92 (62.2)
	T1	128 (36.8)	56 (37.8)
**Pathology grade**	PUNLMP	51 (14.7)	30 (20.3)
	UCC-LG	179 (51.4)	73 (49.3)
	UCC-HG	118 (22.9)	45 (30.4)
**Tumor number**	Single	190 (54.6)	92 (62.2)
	Multiple	158 (45.4)	56 (37.8)
**tumor diameter**	<3cm	207 (59.5)	97 (65.5)
	≥3cm	141 (40.5)	51 (34.5)
**IPPIT**	Yes	150 (43.1)	58 (39.2)
	No	198 (56.9)	90 (60.8)
**NLR**	<2.5	162 (46.6)	82 (55.4)
	≥2.5	186 (53.4)	66 (44.6)
**PLR**	<150	215 (61.8)	93 (62.8)
	≥150	133 (38.2)	55 (37.2)
**SII**	<525	194 (55.7)	90 (60.8)
	≥525	154 (44.3)	58 (39.2)
**Recurrence**	Yes	150 (43.1)	58 (39.2)
	No	198 (56.9)	90 (60.8)

IPPIT, immediate postoperative instillation therapy; PUNLMP, papillary urothelial neoplasms of low malignant potential; UCC-LG, urothelial carcinoma-low grade; UCC-HG, urothelial carcinoma-high grade.

### 3.2 Screening for independent risk factors

The collected potential factors that may be associated with recurrence were included in the univariate regression analysis, which ultimately showed that smoking, tumor stage, pathological grade, number, IPPIT, NLR, PLR, and SII were associated with RFS (all P < 0.05). These factors were then included in the multivariate analysis, which showed that tumor grade, IPPIT, NLR, and SII were independent risk factors for RFS (**Table 2**).

**Table 2:** Factors predicting the recurrence of NMIBC by univariate and multivariate Cox regression analysis (training cohort).

Variable	Univariate analyses			Multivariate analyses		
	Hazard ratio	95% CI	p Value	Hazard ratio	95% CI	p Value
**Smoking history**	1.621	1.006–2.611	0.047	1.455	0.883–2.398	
**Pathology grade**	1.745	1.112–2.739	0.015	1.669	1.036–2.690	0.035
**T category**	1.651	1.046–2.606	0.031	1.187	0.731–1.928	
**Tumor number**	1.594	1.011–2.513	0.045	1.341	0.838–2.146	
**IPPIT**	0.501	0.293–0.855	0.011	0.564	0.322–0.988	0.045
**NLR**	2.573	1.572–4.214	<0.001	1.842	1.017–3.336	0.044
**PLR**	1.796	1.136–2.838	0.012	1.030	0.616–1.722	
**SII**	2.739	1.707–4.395	<0.001	1.905	1.052–3.449	0.033

### 3.3 Construction and verification of the nomogram

**Figure 1:**
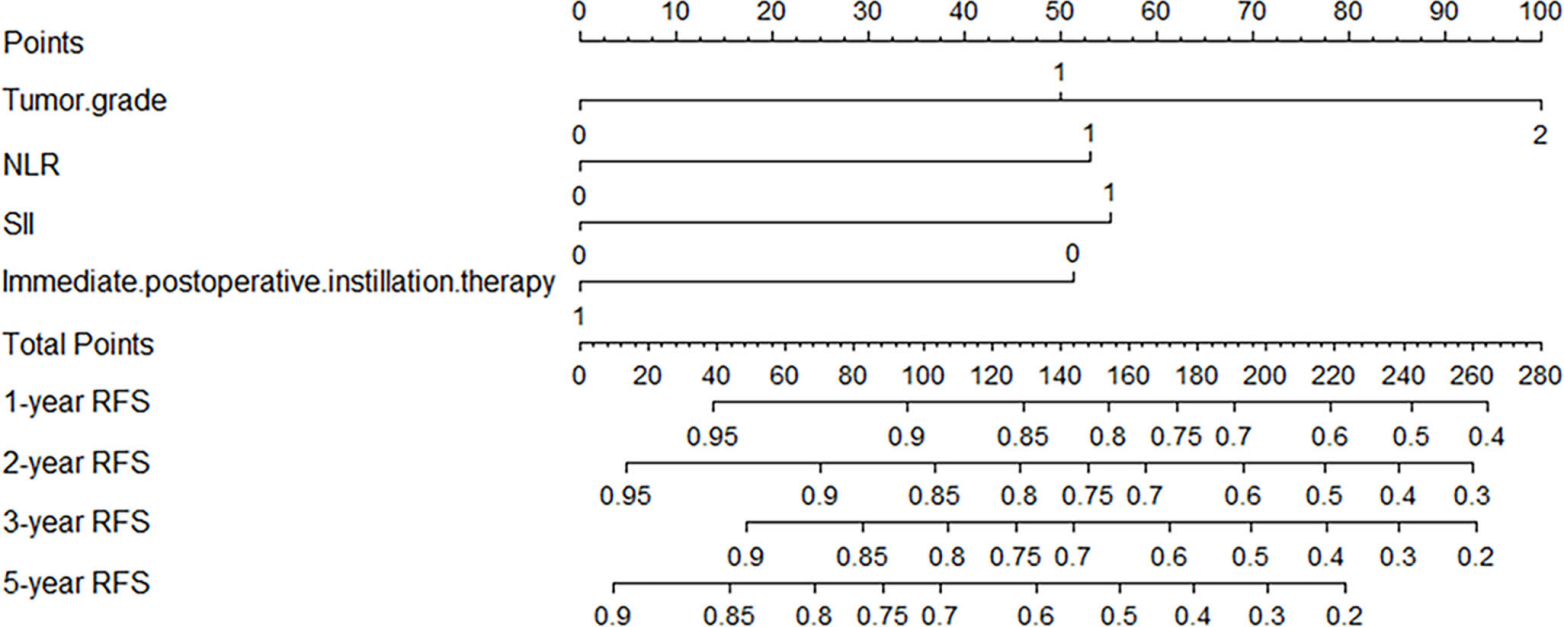
The nomogram model was used to predict RFS at 1, 2, 3, and 5 years after NMIBC resection.

**Figure 2:**
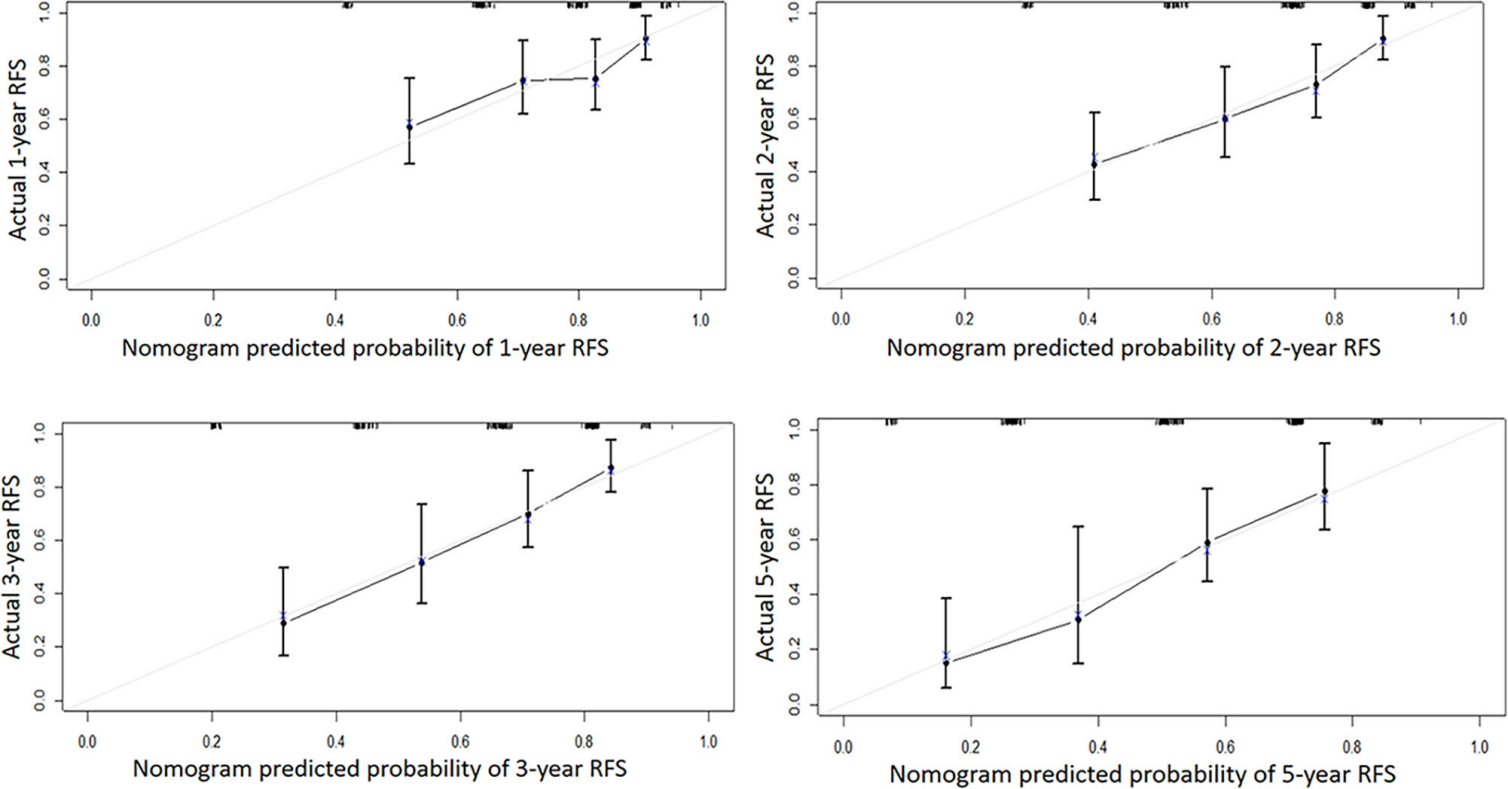
The internal calibration curve of the Nomogram model. Solid line: the prediction curve generated by the Nomogram model. Dashed line: reference line.

**Figure 3:**
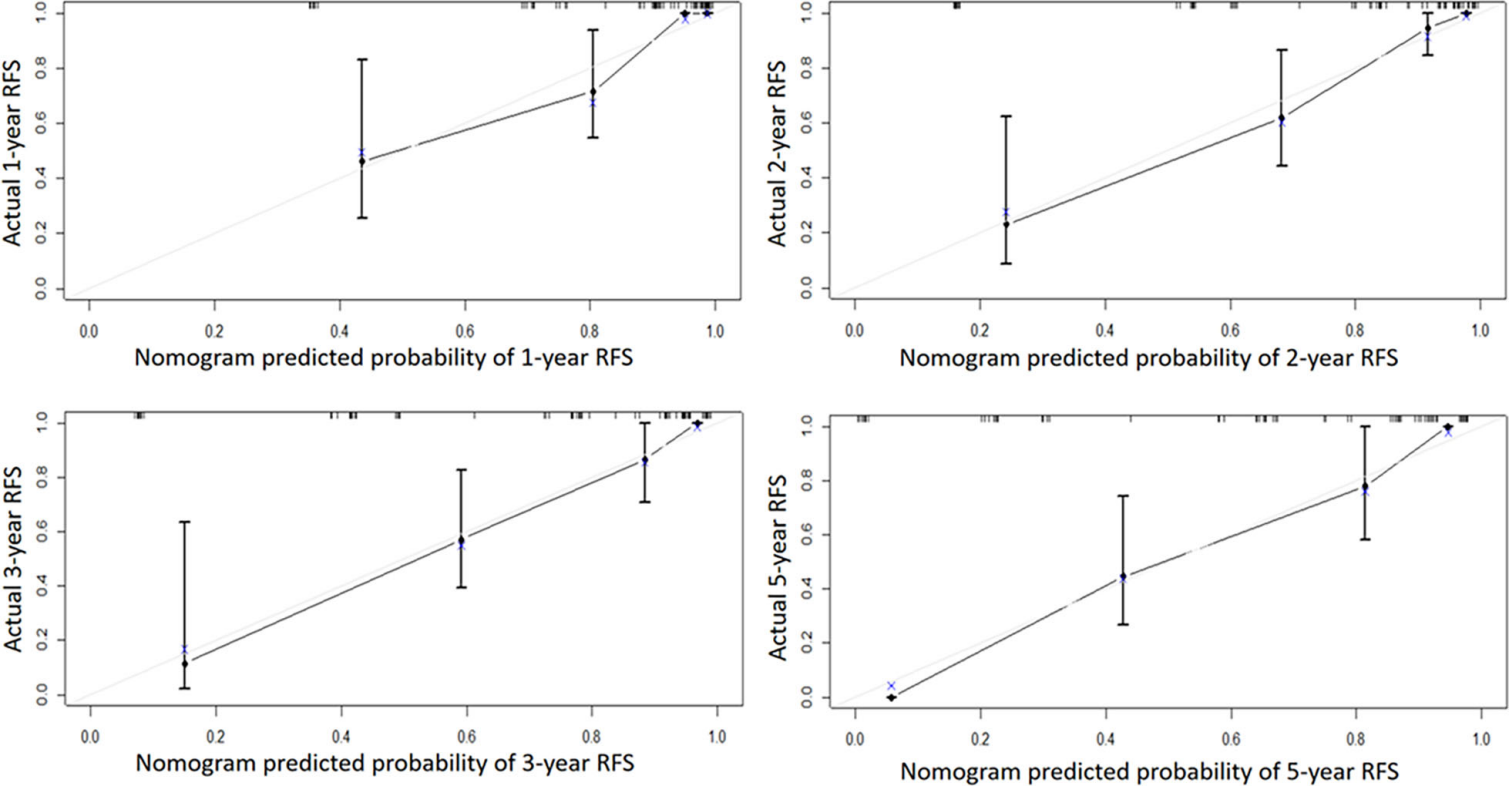
The external calibration curve of the Nomogram model. Solid line: the prediction curve generated by the Nomogram model. Dashed line: reference line.

Independent risk factors affecting RFS in inpatients were included in the plotting of the nomogram, and a nomogram model for predicting RFS in patients with NMIBC at 1, 2, 3, and 5 years was obtained (**Figure 1**). According to the score on the nomogram corresponding to each risk factor for the patient, the total score is obtained by adding it, and then the RFS of the patient for 1, 2, 3, and 5 years can be obtained. In the training and validation cohorts, the internal calibration curves (**Figure 2**) and the external calibration curves (**Figure 3**) demonstrated a good fit. The C-index of the training cohort and the validation cohort were 0.824 (95% CI: 0.77–0.88) and 0.841 (95% CI: 0.78–0.91), respectively. Finally, we collected the C-index of the different models and compared their accuracy in predicting postoperative RFS in NMIBC patients (**Table 3**). The results showed that the C-index of this model was the highest, which represented the high accuracy of the model combining inflammatory response markers with traditional clinical parameters for disease prediction.

**Table 3:** The discriminatory power (C-index) of different models.

Model	Prognostic factors	C-index
**Model 1**	the number of tumors, tumor size, prior recurrence rate, T stage, concomitant CIS, and grade.	0.660
**Model 2**	gender, age, recurrent tumor, number of tumors, T stage, concomitant carcinoma in situ, and grade.	0.636
**Model 3**	age, gender, stage, concomitant CIS, tumor size, and the administration of adjuvant intravesical therapy, preoperative serum DRR.	0.683
**Model 4**	the number of tumors, T stage, smoking history, grade, and IPPIT.	0.652
**This model**	tumor grade, IPPIT, NLR, and SII.	0.824

Model 1, EORTC model (4); Model 2, CUETO model (4); Model 3, DRR model (5); Model 4, Traditional clinical parameter models; DRR, The De Ritis ratio (aspartate aminotransferase/alanine aminotransferase); IPPIT, immediate postoperative instillation therapy; CIS, carcinoma in situ.

### 3.4 Optimal threshold of the nomogram model

**Figure 4:**
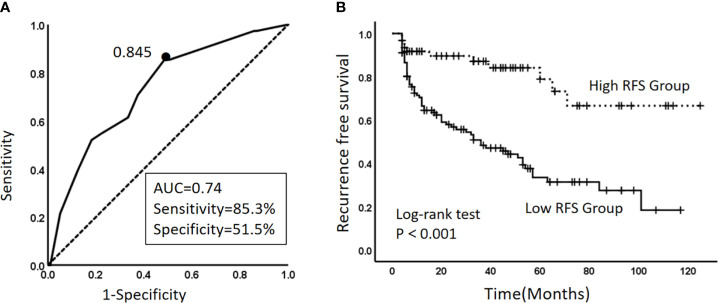
**(A)** Curve of the optimal threshold for the 1-year RFS predicted by the model. The area under the curve at the “black dot” is the largest, which indicates that the optimal threshold for the 1-year RFS predicted by the model is 0.845 (AUC, area under the curve = 0.74; sensitivity 85.3%; specificity, 51.5%) (solid line: ROC curve of the model; dashed line: reference line). **(B)** Kaplan–Meier survival curves for the high-RFS group and the low-RFS group. Dashed line: RFS curve for the high-RFS group. Solid line: RFS curve for the low-RFS group.

In the training cohort, the ROC curve and Youden index indicated that the optimal threshold for the model to predict 1-year RFS was 84.5% (AUC 0.732, 95% CI 0.657–0.807) (**Figure 4A**). In general, an AUC between 0.7 and 0.8 is considered acceptable for predictive power of the model. The patients were divided into two groups based on the calculated optimal threshold, with RFS ≤ 84.5% of patients in the low recurrence-free survival group (Low-RFS group) and RFS> 84.5% in the high recurrence-free survival (High-RFS) group. The median follow-up time was 56(4-125) months, and the median recurrence-free survival was 19(4-117) months in the low-RFS group. The median follow-up in the high-RFS group was 50(4-125) months, and the recurrence-free survival was 36(4-125) months. The 1-year RFS rates were 49.6% (95% CI: 40.2–59.1) and 97.5% (95% CI: 83.1–111.8) in the low and high RFS groups, respectively (P<0.001) (**Figure 4B**).

## 4 Discussion

Previous studies have found that neutrophils in TME release factors associated with tumor proliferation and metastasis, such as tumor necrosis factor (TNF), vascular endothelial growth factor (VEGF), elastase, and matrix metalloproteinases (19, 20). Lymphocytes exert antitumor effects in organisms by inducing lysis and apoptosis of target cells (21). Joseph N found that lymphopenia was an independent prognostic factor for patients with muscle-invasive bladder cancer receiving radical chemoradiotherapy and advanced bladder cancer patients receiving palliative chemotherapy (22). Lymphocytes are closely related to the body’s immunity, and when the number of lymphocytes in the TME decreases, local immunity decreases, which in turn provides a favorable microenvironment for the growth of tumors. Similarly, Platelets release VEGF to promote angiogenesis, enhance tumor cell aggregation, and further promote tumorigenesis and progression (23). A previous study observed bladder cancer patients after radical cystectomy and pelvic lymphadenectomy and showed that preoperative albumin, lymphocyte count, and platelet count were independently associated with a significantly increased risk of death from bladder cancer (24).

In this context, some inflammatory markers based on the above two or three types of cells have been proposed to predict tumor prognosis, such as NLR, PLR, and SII. These inflammatory markers can better reflect the body’s inflammatory response and the dynamic balance of antitumor effects than single inflammatory cells, so they have a certain predictive effect on the prognosis of tumors.

In addition to TURBT, bladder infusion chemotherapy is also an important adjuvant treatment for NMIBC patients. Previously, several studies have reported that postoperative instillation chemotherapy is effective in reducing local tumor recurrence, especially IPPIT, which was more effective in reducing the 5-year recurrence rate of patients based on its ability to promptly kill intraoperatively disseminated tumor cells and residual tumor cells at the trauma site (18, 25). Typically, clinicians choose a postoperative instillation chemotherapy regimen based on the guidelines and the patient’s condition. This study is similar to previous reports and confirms that IPPIT significantly improves patient outcomes.

Pathological grade, IPPIT, NLR, and SII were independently linked with RFS in this study when inflammatory factors were combined with conventional clinicopathological factors. In the end, the combination of the aforementioned elements results in the construction of a nomogram model for predicting postoperative recurrence in NMIBC patients, which may roughly predict the recurrence of patients after surgery. Previous studies have found that NMIBC patients tended to experience recurrence one year after surgery; in this study, the optimal cutoff value of 1-year RFS for patients was calculated using the ROC curve; and the results showed that the 1-year RFS rate was significantly higher in the high RFS group than in the low RFS group (26). Therefore, for the two groups with different oncological outcomes, we can implement individualized clinical interventions and measures for the patients. For example, a patient with high-grade UC (100 points), NLR=3.0 (53 points), SII=665 (55 points), receiving IPPIT (0 points), who had a total score of 208 in the model, had 1-year, 2-year, 3-year, and 5-year RFS of 64%, 54%, 45%, and 26%, respectively (1-, 2-, 3-, and 5-year probability of recurrence of 36%, 46%, 55%, and 74%). For this patient, who has a high possibility of recurrence, we should increase the frequency of follow-up or chemotherapy cycles or even perform radical resection in his/her follow-up treatment. For patients with a low possibility of recurrence, we can use relatively simple postoperative strategies such as reducing the frequency of follow-up visits to improve patients’ quality of life. Through our model, clinicians can even develop a more personalized treatment strategy for each patient.

In recent years, variant histology BC is an emerging topic and several studies have reported their impact on the prognosis of both NMIBC and MIBC. A study analyzed VHs of high-grade T1 (HGT1) bladder urothelial carcinoma and found that it was identified as a significant predictor of DFS. They considered patients with micropapillary, nested, or basaloid morphology or glandular divergent differentiation carcinoma as high-risk HGT1, while the presence of divergent squamous differentiation, inverted growth, microcystic, and villouslike or lymphoepithelioma-like carcinoma was considered as low-risk HGT1 (15). Francesco Claps’ team evaluated VHs for disease-specific survival (DSS) in patients with invasive urothelial BC undergoing radical cystectomy (RC). They found that clear-cell, plasmacytoid, small-cell and sarcomatoid VHs were associated with worse DSS compared to pure UC (27). In this study, we used VH as an exclusion criterion, considering the differences in reporting criteria by pathologists at different institutions and the small number of relevant cases. At present, some molecular markers, such as lncRNA, urine cell-precipitated DNA samples, and YAP 1 activation are used in the diagnosis, prognosis, and treatment of bladder cancer (28), but they have not been applied in clinical practice. A study created a Controlling Nutritional Status (CONUT) scoring system combining albumin, lymphocyte count, and total cholesterol. This nutrition-related marker was independently associated with a poorer postoperative course in BC patients treated with RC and predicted worse RFS, OS, and CSS (29). We have combined traditional clinical parameters and readily available hematological indicators to construct a model that not only effectively predicts disease recurrence but also reduces the burden on patients.

Of course, this study had certain limitations. First, some of our limitations are inherent in retrospective and multicenter studies, with surgeon experience and treatment strategies varying from institution to institution. Second, due to the presence of telephone follow-up, the specific situation of recurrence or progression is not yet known, so this study does not distinguish between tumor recurrence and progression. Additionally, we did not collect some nutrition-related markers and biomarkers, and there are currently no standardized cutoff points for inflammatory markers. Despite our exclusion criteria for patients with VHs, the fact remains that some pathologists do not recognize or report about one-half of cases with variant histology in their practice (15). Therefore, the risk associated with variant histology in BC might indeed be underrecognized, which is one of our limitations. Finally, we did not further analyze the relationship between inflammatory factors and pathological parameters. Another study found a causal relationship between p53 loss and cancer-induced systemic neutrophil inflammation (30). In a later phase, we will further explore the relationship between inflammatory factors and tumor-related markers.

## 5 Conclusion

In this study, a nomogram model constructed with inflammatory markers and traditional clinical parameters effectively predicted RFS after TURBT in patients with NMIBC. Based on this model, clinicians can provide patients with more ideal personalized treatment plans after surgery to improve patients’ quality of life.

## Data availability statement

The original contributions presented in the study are included in the article/supplementary material. Further inquiries can be directed to the corresponding author.

## Ethics statement

The studies involving human participants were reviewed and approved by Medical Ethics Committee of Yongchuan Hospital of Chongqing Medical University. Written informed consent for participation was not required for this study in accordance with the national legislation and the institutional requirements.

## Author contributions

Conceptualization, XY and TZ. Formal analysis, YX and WF. Methodology, XY, JP, JL and TZ. Resources, JL and LW. Software, XY and JP. Validation, JP and CL. Writing – original draft, All authors; Writing – review and editing, CL and TZ. All authors contributed to the article and approved the submitted version.
